# Renaturing Membrane Proteins in the Lipid Cubic Phase, a Nanoporous Membrane Mimetic

**DOI:** 10.1038/srep05806

**Published:** 2014-07-24

**Authors:** Dianfan Li, Martin Caffrey

**Affiliations:** 1 School of Biochemistry and Immunology & School of Medicine, Trinity College Dublin, Dublin, Ireland

## Abstract

Membrane proteins play vital roles in the life of the cell and are important therapeutic targets. Producing them in large quantities, pure and fully functional is a major challenge. Many promising projects end when intractable aggregates or precipitates form. Here we show how such unfolded aggregates can be solubilized and the solution mixed with lipid to spontaneously self-assemble a bicontinuous cubic mesophase into the bilayer of which the protein, in a confined, chaperonin-like environment, reconstitutes with 100% efficiency. The test protein, diacylglycerol kinase, reconstituted in the bilayer of the mesophase, was then crystallized *in situ* by the *in meso* or lipid cubic phase method providing an X-ray structure to a resolution of 2.55 Å. This highly efficient, inexpensive, simple and rapid approach should find application wherever properly folded, membrane reconstituted and functional proteins are required where the starting material is a denatured aggregate.

Most human diseases, not involving infection, progress due to protein misfolding. Cystic fibrosis, cancers, neurodegenerative diseases, amyloidoses, cataracts and sickle cell anaemia are prime examples^1^. Protein folding is the assumption by a linear polypeptide of a 3D form that can engage in functional interactions. Getting it right is a delicate balancing act with mere kTs in favour of the correctly folded form^2^. In contrast to their soluble counterparts, membrane proteins must navigate a particularly complex folding journey; its extra-membrane domains must conform to the dictates of a crowded aqueous space while its membrane integral parts adjust to the varied physical and chemical profiles that exist along and across the membrane plane^3^. It is not surprizing then that homologous and heterologous overexpression and cell-free production of membrane proteins is experimentally challenging, and instead of a properly folded and functional protein, often one ends up with a precipitate in the form of an aggregate or an inclusion body. Renaturing the precipitated protein is an option. This could involve an initial solubilization in a denaturing solvent followed by refolding and reconstitution into one of several membrane mimetics^4^,^5^,^6^. The simplest, but most unnatural of these is the detergent micelle^7^,^8^,^9^. Others include amphipols^14^, nanodiscs^10^ and bilayered vesicles^11^,^12^,^13^. Amphipols are synthetic amphiphilic polyacrylates that vary in composition, charge and molecular size. Despite bearing little resemblance to a natural membrane, they are relatively efficient (~50%) in solubilizing membrane proteins. As expected, yield rises when the amphipol is augmented with membrane lipids. Nanodiscs are composites that include a scaffolding protein and bilayer forming phospholipids. They have been used successfully to functionally reconstitute membrane proteins. For end uses such as crystallization, the nanodisc lipid and scaffold protein must be removed which makes them a little more challenging to work with. Vesicles or liposomes are close to ideal membrane mimetics for refolding and reconstitution. However, yields can be low and protein concentration in the final membrane is often not high enough for downstream applications like crystallogenesis. The current work focuses on a vogue mimetic, the lipid cubic phase^14^ which affords highly efficient renaturation into a lipid membrane ready for use in myriad downstream biochemical and biophysical applications, including crystallogenesis.

The lipid cubic phase is a bicontinuous, lyotropic liquid crystal or mesophase with myriad applications. The more impressive of late is as a medium for crystallizing membrane proteins. This has provided the structural and functional biology community with many structures of scientifically and medically important membrane proteins that feed into drug discovery programs^14^,^15^. The mesophase is composed of a continuous, curved bilayer on either side of which is a multiply branched, continuous aqueous channel. It is an attractive bicontinuous medium in which to grow crystals of membrane proteins because the process takes place in a membrane mimetic where proteins have been shown to be folded and functional^16^,^17^,^18^.

The lipids used to make the cubic phase are the monoacylglycerols, monoolein being the most popular. When combined in equal parts with water, monoolein forms the cubic phase which is stable from 18 to 90 °C^19^. Fully hydrated, it can exist in equilibrium with bulk water. The cubic phase incorporates an extraordinarily high density of membrane; a gram of cubic phase has a membrane/aqueous interface of ~350 m^2^ (Ref. 20). As a bicontinuous mesophase, it is highly porous reminiscent of a molecular sponge. This means that water-soluble materials of appropriate sizes can diffuse in and out of a bolus bathed in aqueous solution. The cubic phase is sticky and viscous, like thick toothpaste. Whilst challenging in certain applications, this hallmark rheology is a boon in others. Usefully, the cubic phase is compatible with additives that range from detergents to salts and denaturants that include urea and guanidinium chloride^21^.

In the course of developing the cubic phase as a medium for high-throughput crystallization, the above features of the cubic phase suggested an application for refolding denatured integral membrane proteins. Being able to solubilize, renature and to functionally reconstitute membrane proteins in a lipid bilayer is very attractive. Here, we describe a method for doing just that with 100% efficiency beginning with a membrane kinase enzyme as a detergent- and phospholipid-depleted precipitate. The salvaged protein recovered full catalytic activity and went on to produce crystals *in meso* and a 2.55 Å structure identical to that of the untreated, reference kinase^22^.

## Results

### The hypothesis

**Figure 1:**
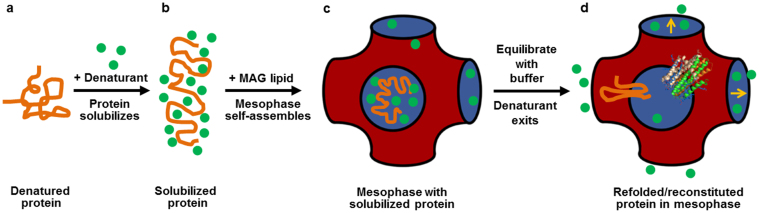
Renaturing a membrane protein in the lipid cubic phase. Precipitated protein (a) is solubilized in a denaturing solvent (b). The solubilized protein solution is combined with monoacylglycerol lipid to spontaneously form the cubic mesophase (c). Equilibrating the mesophase with denaturant-free buffer enables denaturant to preferentially exit the nanoporous channels and the protein to spontaneously reconstitute into the adjacent bilayer driven by the falling denaturant concentration (d). The bicontinuous mesophase creates a confining, chaperonin-like environment that mitigates aggregation and a receptacle into which the protein reconstitutes and subunits assemble.

The renaturation method makes use of the lipid cubic phase and is based on the following hypothesis (Fig. 1). The aggregated and unfolded protein, solubilized in a concentrated denaturing aqueous solvent such as acidic urea, will form the cubic phase by spontaneous self-assembly when mixed with monoolein. Upon bathing in an excess of denaturant-free buffer, the denaturant will diffuse out of the protein-laden mesophase which acts like a nanoporous molecular sponge. The solubilized protein within the confined, nanometer-sized aqueous channels will respond to the falling concentration of denaturant and to a drop in its solubility by reconstituting into the bilayer of the mesophase in its immediate vicinity, as opposed to aggregating. As the denaturant concentration continues to fall, the intra- and extra-membrane domains of the protein will renature. And protomers, as appropriate, will reassemble to generate a reconstituted and functional protein ready for immediate use in downstream applications such as biochemical and biophysical characterization and crystallogenesis.

### Denatured test protein

**Figure 2:**
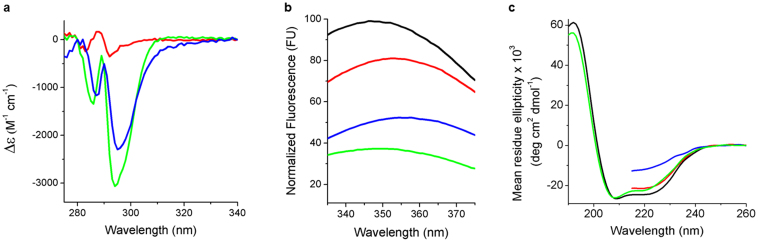
Spectroscopic evidence for a denatured kinase. (a), UV difference spectra. The reference spectrum was recorded with untreated DgkA in detergent micelles. Sample spectra, from which the reference spectrum was subtracted, were recorded with detergent-free DgkA* in SDS (green), in acidic urea (blue), and DgkA in acidic urea containing detergent (red). The minimum at 294 nm is characteristic of denatured DgkA^28^. (b), Fluorescence emission of untreated DgkA in detergent micelles (black) and in acidic urea containing detergent (red), and of detergent-free DgkA* in SDS (green) and in acidic urea (blue). A red shift, coupled with a reduction in fluorescence yield, is indicative of tryptophan exposure to a polar environment and to denaturation. (c), Circular dichroism spectra of untreated DgkA in detergent micelles (black) and in acidic urea containing detergent (red), and of detergent-free DgkA* in SDS (green) and in acidic urea (blue). In urea, data are only shown to 215 nm due to strong absorbance at shorter wavelengths. A strong negative ellipticity at 222 nm correlates with α-helical secondary structure and the folded state. All data shown were collected with thermostable DgkA. The corresponding data for wild-type DgkA (Supplementary Fig. S2) show that both constructs behaved similarly.

**Figure 3:**
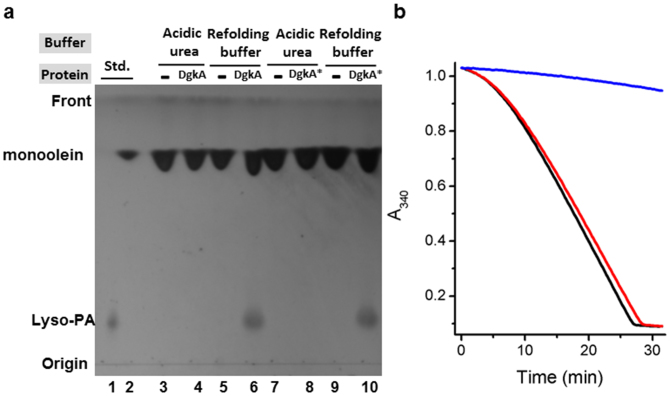
DgkA* renatured in the cubic phase is fully functional. (a), Thin layer chromatographic analysis shows that equal amounts of product, lysophosphatidic acid (lyso-PA), were formed after 1 hour of reaction catalyzed by untreated DgkA (lanes 5, 6) and by detergent-free DgkA* (lanes 9, 10) renatured from acidic urea with denaturant-free refolding buffer. No reaction was seen when acidic urea remained in the mesophase (lanes 3, 4 and lanes 7, 8). Lipid standards are in lanes 1 and 2. (b), Kinase progress curves for untreated DgkA (black) and detergent-free DgkA* (red) renatured from acidic urea. A protein-free control is shown (blue).

**Figure 4:**
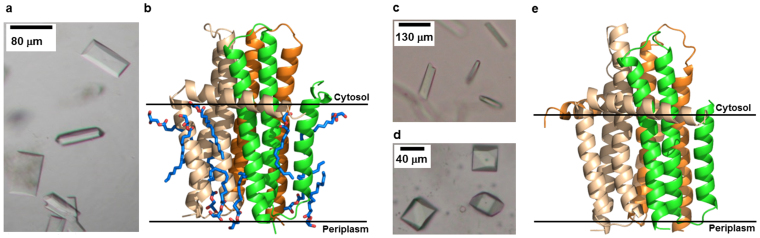
Crystals and structure of refolded kinase. (a), Crystals of thermostable DgkA* grown from the cubic phase into which the protein had been renatured from solubilized precipitate and whose concentration in the mesophase had been increased by multiple rounds of reconstitution. (b), Structure of renatured thermostable DgkA* obtained using crystals like those in (a) to a resolution of 2.55 Å. The protein (ribbon model) is displayed with chains A, B and C coloured wheat, green and orange, respectively. The structure includes 10 lipids (stick models). (c), Monoolein (9.9 MAG) or (d), 7.8 MAG was used as host lipid for refolding and crystallization of wild-type DgkA*. Crystals in (c) and (d) diffracted to 4 Å and 3.8 Å, respectively. For comparison, wild-type DgkA, reconstituted from DM solution into the cubic mesophase, with monoolein or 7.8 MAG as host lipids, both diffracted to 3.7 Å^22^,^32^. (e), Structure of wild-type DgkA* obtained using crystals from the 7.8 MAG mesophase like those in (d) to a resolution of 3.8 Å.

To test the renaturation hypothesis, an integral membrane protein as fully denatured as possible was required. Ideally, the protein should have a sensitive functional assay with which to track its reconstitution and renaturation. DgkA is a homo-trimeric kinase involved in phospholipid metabolism in Gram negative bacteria^23^. The smallest known kinase, it consists of four helices, three of which are either fully or partly membrane embedded while the fourth resides at the membrane surface^22^. The structure of a wild-type and a fully functional, thermostable variant was determined recently to 3.7 Å and 2.05 Å, respectively, using crystals grown *in meso*^22^. Having long served as a model for studies of membrane protein folding^7^,^13^,^24^,^25^, assembly^26^,^27^ and stability^28^,^29^, DgkA was chosen as the test protein. The two DgkA constructs gave almost identical results in this study (Fig. 2,3,4, Supplementary Fig. S2–S3). In the interests of brevity and clarity therefore the focus here is on the thermostable variant.

To begin, a denatured form of the enzyme was generated by treatment with trichloroacetic acid (TCA). The protein had been purified using n-decyl-β-D-maltoside (DM). To minimize the possibility of there being residual detergent in which the protein could retain a native state, the precipitated enzyme was washed extensively (see Methods). The final preparation contained less than 0.1 and 0.3 moles of detergent and phospholipid, respectively, per mole of DgkA monomer. Henceforth, an asterisk will be used to identify this detergent/phospholipid depleted preparation (DgkA*). DgkA (without the asterisk) refers to the folded enzyme in DM micelles. It was possible to solubilize the precipitated and washed DgkA* in 8.5 M urea to the extent of 0.1 mg/mL. For certain applications, a higher protein concentration was needed. Accordingly, the urea was supplemented with 2%(v/v) formic acid which raised solubility to 1 mg/mL.

The extent of denaturation in acidic urea was quantified by monitoring changes in the electronic excitation and emission characteristics and circular dichroic (CD) behaviour of the protein. Absorbance, due to aromatic residues in the 270–340 nm range, is sensitive to denaturation with signature changes in extinction coefficient at 294 nm (Δε). Consistent with the literature^28^, the effect of the harsh detergent, sodium dodecylsulfate (SDS), on DkgA* was to produce a large Δε corresponding to protein denaturation (Fig. 2A). A similar effect, albeit of lesser magnitude, was seen with acidic urea. Interestingly, when the enzyme was dispersed in DM micelles, the acidic urea-induced change was considerably less, suggesting the micelle played a protective role. These data indicate that acidic urea is an effective denaturing solvent. To greatest effect however, it should be used with detergent-depleted protein.

The protein's tryptophan fluorescence yield was dramatically reduced in SDS (Fig. 2B). This is consistent with exposure of DgkA's five tryptophans to a more polar environment, as expected upon denaturation. Acidic urea had a similar although lesser effect to SDS. And, as noted for absorbance, DM micelles appeared to protect lessening the impact of denaturation.

The fact that DgkA is predominantly α-helical is reflected in its CD spectrum (Fig. 2C). The mean residue ellipticity at 222 nm translates to an α-helical content of 70%^30^, close to the crystal structure value of 74%. In SDS, the α-helical content fell by 6% suggesting that this harsh detergent does not profoundly affect the protein's secondary structure. However, in acidic urea the helical content dropped to 36%. Again DM micelles, when present, protected the protein which ended with a residual α-helical content of 56% in denaturant.

An inspection of the crystal structure reveals that the α-helical membrane embedded domain of DgkA accounts for 44% of the total structure. Clearly, the CD results suggest that in denaturant, where the residual helical content has dropped to 36%, all of the extra-membrane and some of the intra-membrane domain has lost its ordered secondary structure.

Together, these spectroscopic data support the view that DgkA* in acidic urea is in as denatured a state as we can get it, and yet still be usable for evaluating the renaturation hypothesis.

### Renaturation in the lipid cubic phase

The renaturation hypothesis was tested by mixing monoolein with a solution of denatured DgkA* dissolved in acidic urea and then bathing the spontaneously-assembled mesophase in denaturant-free buffer. DgkA is a promiscuous enzyme and, in addition to using diacylglycerol as a substrate, it can also phosphorylate the mesophase forming monoolein^18^. Therefore, the kinase activity of DgkA, as an indicator of reconstitution and renaturation, was conveniently assayed directly *in situ* by adding Mg^+2^-ATP to the solution bathing the mesophase and monitoring, by thin layer chromatography (TLC), the formation of the lipid product, lysophosphatidic acid (lyso-PA). The results show that the protein behaved as expected (Fig. 3a). Lyso-PA, generated by renatured DgkA*, was present to the same extent as observed with untreated enzyme.

Lyso-PA is generated by DgkA in a reaction where ATP is consumed and ADP is produced. The generation of ADP can be coupled to the oxidation of NADH by pyruvate kinase and lactate dehydrogenase^31^ to conveniently monitor kinase activity in the lipid cubic phase^18^. This indirect, coupled spectroscopic assay was implemented here and demonstrated that the renatured and reference kinases were equally active with specific activities of 16.6 ± 0.3 U/mg (1 U = 1 μmol min^−1^) and 17.1 ± 0.2 U/mg, respectively (Fig. 3b). This finding is consistent with a renaturation efficiency by the cubic phase method of 100%. By contrast, DgkA refolded by rapid dilution from acidic urea (1% formic acid in 6.5 M urea) into dioleoylphosphatidylcholine vesicles and DM micelles with efficiencies of 26 and 46%, respectively^7^. Thus, the simple process of mesophase formation and bathing with excess denaturant-free solvent facilitated a transformation from unfolded kinase monomers in denaturant solution to membrane integral, refolded and properly assembled trimers with full enzymatic activity.

### Renatured kinase yields native crystal structure

The direct and coupled kinase assays demonstrated convincingly that the renatured protein was fully functional. To prove it was also valuable for downstream biophysical characterization, including 3D structure determination, its ability to produce structure-quality crystals was explored. *In meso* crystallogenesis was the method of choice because the protein, post-renaturation, already resided in the cubic mesophase and, as noted, DgkA crystallizes *in meso*. A DgkA solution at 12 mg/mL, corresponding to an overall protein concentration in the fully hydrated mesophase of ~5 mg/mL, is required for crystallization. As recorded above, DgkA* had been solubilized in denaturing solvent at 1 mg/mL which translates to 0.4 mg/mL protein in the mesophase. This is 12-fold less than the concentration required for crystal growth. The fact that the cubic phase is porous and bicontinuous and that it exists in equilibrium with bulk aqueous medium suggested a way in which the protein load within the mesophase could be raised. This involved multiple rounds of reconstitution where dilute protein solution was used, first to form the mesophase and subsequently to augment the protein complement of the mesophase membrane through the process of partitioning (Supplementary Fig. S1). Repeating the reconstitution process several times, the concentration of protein in the mesophase could be raised to where it was high enough to proceed with crystallization. The beauty of this approach is that the excess, protein-depleted aqueous solution that separates naturally from the fully hydrated mesophase can be drawn off and replaced with fresh protein solution to continue the augmentation process or with a small amount of lipid to produce the pure, optically clear cubic mesophase for use in crystallogenesis.

The rounds of reconstitution protocol just described was implemented and crystallization trials were set up following standard methods. Convincingly, the refolded protein produced crystals that provided a 2.55 Å structure by molecular replacement (Fig. 4, Supplementary Table S1). The structure was virtually identical (RMSD, 0.348 Å) to that observed with untreated reference thermostable DgkA. Likewise, the refolded wild-type DgkA* was crystallized *in meso* using 9.9 MAG or 7.8 MAG as host lipids (Fig. 4c,d). Crystals in 9.9 MAG diffracted to 4 Å. A complete data set to 3.8 Å (Supplementary Table S1) was obtained with crystals grown in 7.8 MAG and was used for structure determination. The structure of the refolded wild-type DgkA* is virtually identical (RMSD, 0.419 Å) to the 3.7 Å structure recorded for the untreated, reference wild-type enzyme.

## Discussion

The hypothesis tested and proven here provides for a highly efficient, simple, inexpensive and rapid means for transforming useless precipitate into a fully functional, bilayer-reconstituted integral membrane protein for use in biochemical and biophysical studies. The end product can be employed for direct *in meso* crystallization for high resolution 3D structure determination. The rounds of reconstitution approach should prove valuable for applications requiring high concentrations of membrane integrated proteins that include crystallogenesis and vaccine development. These results highlight the bicontinuous cubic phase as an extremely versatile and useful nanoporous membrane mimetic that can double as a chaperonin to mitigate aggregation and as a receptacle into which the membrane protein can renature.

## Methods

DgkA purification, enzymatic assays (spectroscopic and chromatographic), and *in meso* crystallization were carried out as described^18^,^32^. Detergent and lipid concentrations were assayed as detailed in Supplementary Methods. DgkA was unfolded by precipitating in TCA, rendered ‘detergent-free’ by washing seven times with water, and then solubilized in SDS or acidic urea. Unfolding was monitored by absorbance, fluorescence and CD. Refolding was done by combining monoacylglycerol (MAG) with solubilized DgkA* to form the cubic phase and then incubating the mesophase with denaturant-free buffer in 96-well plates or Eppendorf tubes for kinase assays and in coupled syringes for crystallization.

DgkA* was soluble in acidic urea to the extent of ~1 mg/mL, 10–20 times below the optimal value for crystallization. A ‘rounds of reconstitution’ approach (Supplementary Methods) was used to enrich DgkA* in the cubic phase. Briefly, a total of 200–320 μL DgkA* at 1 mg/mL was mixed with 24 μL MAG in several rounds of reconstitution, each with 50–60 μL DgkA* solution. The protein preferentially partitioned into the cubic phase and excess protein-free solution, which naturally separates from the mesophase, was removed and was replaced with denaturant-free buffer used to dilute and wash out the denaturant. Excess denaturant-free buffer was replaced with fresh DgkA solution and the reconstitutional homogenization repeated. The process of reconstitution and washing was repeated 3–5 times. The final step was to absorb the small amount of remaining denaturant-free buffer by homogenizing in 2-3 μL MAG which converted the mix to the optically clear cubic phase ready for use in crystallization trials (Supplementary Methods).

X-ray diffraction data were collected at beamlines 23-ID-B (Advanced Photon Source), I24 (Diamond Light Source) and PX II (Swiss Light Source). Details of diffraction data collection and processing, and structure determination and refinement are described under Supplementary Methods.

## Author Contributions

D.L. designed and performed the experiments, analysed data and wrote the manuscript. M.C. formulated the hypotheses, analysed data and wrote the manuscript.

## Additional Information

**Accessions Codes** Atomic coordinates and structure factors for the in meso refolded DgkA* structure are deposited in the Protein Data Bank (accession codes 4BRB for the thermostable mutant, and 4UP6 for the wild type).

## Supplementary Material

Supplementary Information Supplementary Methods, Table S1 and Figure S1-S3

## Notes

****
