# Supplementary material for: Renaturing Membrane Proteins in the Lipid Cubic Phase, a Nanoporous Membrane Mimetic
Source: Sci Rep. 2014 Jul 24;4:5806. doi: 10.1038/srep05806 (PMC4108929; doi:10.1038/srep05806)
Supplement: Supplementary Information — Supplementary Methods, Table S1 and Figure S1-S3 [file srep05806-s1.pdf]

Renaturing Membrane Proteins in the Lipid Cubic Phase, a Nanoporous Membrane Mimetic

Dianfan Li, Martin Caffrey*

School of Biochemistry and Immunology & School of Medicine, Trinity College Dublin, Dublin, Ireland.

* Correspondence email: martin.caffrey@tcd.ie

Supplementary Information

Table of Contents

Supplementary Methods

Table *S1*

Figure *S1-S3*

References

SUPPLEMENTARY METHODS

DgkA protein production. Wild type (WT) DgkA and a thermo-stable mutant (Δ7) (seven changes relative to WT: A41C, C46A, I53V, I70L, M96L, V107D, C113A) were overexpressed and purified, as described[1, 2]. For residual phospholipid analysis, the phosphorous-containing TCEP in the gel filtration buffer[1] was replaced with DTT (1 mM DTT, 0.25 %(w/v) DM, 0.1 M sodium chloride, 10 mM Tris HCl pH 7.8). Protein purified in this manner had no detectable phospholipid using a sensitive colorimetric assay (see below).

Detergent assay. Detergent in the protein sample was quantified using a colorimetric assay, as described[3]. A standard curve was created by adding 90 μL of concentrated sulfuric acid to 6 μL of solutions containing 0.005 – 0.08 %(w/v) decyl-maltoside (DM) in 1.5 mL Eppendorf tubes. After mixing thoroughly, 18 μL of 5 %(w/v) phenol was added, the samples were incubated for 5 min at 90 °C and cooled to room temperature (RT, 20-21 °C). A_{490} of the solution was measured in a plate reader (Molecular Devices SpectraMax M2[e], Sunnyvale, CA, USA) using 0.11 mL samples in a 384-well plate. For TCA-precipitated DgkA samples, the pellet from 8 μL of a 12 mg DgkA/mL solution, resuspended in 6 μL milliQ-water, was used for assay. Measurements were done in triplicate.

Phospholipid assay. The phospholipid content of protein samples was quantified using a published protocol[4] with slight modifications. Briefly, potassium dihydrogen phosphate standards (0 - 1 µg phosphorus), 5 µg of dioleoylphosphatidylcholine and 120 µg test protein (either directly after gel filtration or as a washed pellet, see below) were transferred to 6 mL glass tubes pre-washed with 1 %(v/v) nitric acid. Samples were dried in an oven at 50-60 °C for 10 min. Wet ashing was carried out by adding 0.13 mL 70 %(v/v) perchloric acid followed by incubation in a heat block at 180 °C for 50 min. Upon cooling to RT, 0.66 mL milliQ-water, 0.1 mL 2.5%(w/v) ammonium molybdate and 0.1 mL 10 %(w/v) L-ascorbic acid were sequentially added to each sample with mixing. Colour development was performed by boiling the samples for 5 min. After cooling to RT, 0.33 mL of each sample was placed in 96-well plates and A_{810} was measured in a plate reader (Molecular Devices SpectraMax M2[e]). All samples were assayed in triplicate.

Removing detergent from DgkA. All procedures were carried out at RT. To begin the process of removing detergent, the protein (WT, Δ7) was precipitated by adding 8 μL DgkA (12 mg/mL) to 1.5 mL of 15 %(w/v) TCA. After centrifuging for 10 min at 20,000 g, the pellet was resuspended and washed in 1.5 mL MilliQ-water by alternating vortex mixing and aspirating with a 0.2 mL pipetting device for 5 min. The insoluble pellet was collected by centrifugation for 10 min at 20,000 g and the resuspending/washing procedure was repeated seven times. The final pellet was resuspended in water and the suspension was used for detergent and phospholipid assay, as above. For use in solubilisation and subsequent refolding assays, the washed pellet was air dried overnight at RT. The protein sample, so prepared, was shown to contain no detectable detergent or phospholipid and is identified with an asterisk (DgkA*).

Solubilizing DgkA*. Detergent-depleted DgkA* pellets were solubilized either in **SDS Buffer** (1 mM TCEP, 0.1 M NaCl, 2 %(w/v) SDS, 50 mM HEPES pH 7.5) at RT or in **Acidic Urea Solution** (1 mM TCEP, 0.1 M NaCl, 2 %(v/v) formic acid, 8.5 M urea) at 37 °C by shaking on a thermomixer (800 rpm, Eppendorf Comfort) for 16-20 h, followed by water-bath sonication (Branson 2510) for 30 min at 37 °C. Unsolubilized material was removed by centrifugation at 20,000 g for 10 min at RT.

Coupled kinase assay. DgkA activity was measured by monitoring the oxidation of NADH through a coupled assay involving pyruvate kinase (PK) and lactate dehydrogenase (LDH)[5]. For DgkA purified in DM, the procedure was exactly as reported[1]. Briefly, the protein at 66 μg/mL was reconstituted into the cubic phase by mixing 2 volumes of protein with 3 volumes of monoolein. Five microliters of the protein-laden mesophase was placed in 96-well plates. Kinase activity was initiated by adding 0.2 mL **Assay Buffer** (20 mM ATP, 0.1 mM EDTA, 0.1 mM EGTA, 55 mM magnesium acetate, 1 mM PEP, 0.2 mM DTT, 50 mM LiCl, 0.4 mM NADH, 20 U / mL of PK and LDH, and 75 mM PIPES pH 6.9) pre-warmed to 30 °C. A_{340} was monitored for 30 min at 15 s intervals. For DgkA* solubilized in **Acidic Urea**, the protein-laden mesophase was made in the same manner as above. The kinase assay was performed after bathing the mesophase *in situ* with 0.2 mL **Refolding Buffer** (1 mM TCEP, 0.1 M NaCl, 0.1 M HEPES pH 7.5) for 30 min at RT to wash out denaturants. The bathing solution was removed by inverting the plate onto tissue paper and the process repeated twice. After carefully removing the bathing buffer, Assay Buffer was added and the kinase measurement was performed, as above. Negative controls lacked protein. Assays were run in triplicate.

Direct, chromatographic kinase assay. To measure kinase activity *in meso* in **Acidic Urea**, the formic acid and urea components of which were incompatible with PK and LDH in the coupled assay above, the direct ATP-dependent production of lyso-phosphatidic acid (lyso-PA) from monoolein was monitored using TLC[1]. Typically, 20 μL of the protein-laden mesophase in a 1.5 mL Eppendorf tube was incubated at RT with 1 mL of either **Acidic Urea** or **Refolding Buffer**. After shaking (800 rpm, Thermomixer) for 30 min at RT, the bathing solutions were replaced with fresh bathing solution, and the process repeated twice. The kinase reaction was initiated by adding to the bathing solution magnesium acetate and ATP to 10 mM and 60 mM, respectively. The reaction was allowed to run for 1 h at 30 °C with shaking (800 rpm, Thermomixer) after which the bathing solution was removed and 0.8 mL chloroform:water (1:1 by vol.) was added to solubilize the lipid. Centrifugation at 20,000 g for 10 min at RT separated the phases and the upper aqueous phase was removed. 2 μL of the chloroform solution containing the extracted lipid were loaded onto TLC plates (Cat. No. 1.05554.0001, HX068423. Merck, Darmstadt, Germany) that had been pre-run in chloroform. The plates were developed in chloroform:methanol:acetone:acetic acid:water (10:2:4:2:1 by vol.) at RT. After drying on a heat block at 40 °C under nitrogen for 15 min and staining with 20 %(w/v) phosphomolybdic acid in ethanol, the plate was placed on a hot plate at 150 °C for stain development.

Absorption and fluorescence spectroscopy. All spectrophotometric measurements were carried out at RT. Four samples each for WT and Δ7 DgkA were used. i) Protein solubilized in **DM detergent**, ii) Protein solubilized in DM added to **Acidic Urea with Detergent** (0.25 %(w/v) DM, 1 mM TCEP, 0.1 M NaCl, 2 %(v/v) formic acid, 8.5 M urea), iii) Detergent-free DgkA* solubilized in **SDS Buffer** (1 mM TCEP, 0.1 M NaCl, 2 %(w/v) SDS, 50 mM HEPES pH 7.5), and iv) Detergent-free DgkA* solubilized in **Acidic Urea Buffer** (1 mM TCEP, 0.1 M NaCl, 2 %(v/v) formic acid, 8.5 M urea). Spectra were baseline corrected using protein-free buffers. Measurements were performed in triplicate.

Protein concentration was determined by measuring A_{280} ($\varepsilon_{1\ mg/ml} = 2.1$)[5, 6] in a NanoDrop 1000 spectrometer (Thermo Fisher Scientific Inc., Wilmington, DE). UV-visible spectroscopic analysis was performed at 0.64 mg protein/mL in a 1 cm-pathlength quartz cuvette (Sigma Aldrich, St. Louis, MO, USA) with a UVIKON XL spectrophotometer (Northstar Scientific, Leeds, UK). Spectra were recorded from 340-275 nm at a scanning speed of 200 nm/min. For comparisons, all spectra were scaled to the same A_{280}. Difference spectra were obtained by subtracting the spectrum recorded in **DM** from that recorded in **Acidic Urea with Detergent**, **SDS Buffer** and **Acidic Urea**.

Fluorescence measurements were carried out at 0.1 mg protein/mL in a 3 mm pathlength quartz cuvette (Hellma, Jena, Germany) with a FluoroMax-3 spectrofluorometer (Horiba, Kyoto, Japan). Emission spectra were recorded from 375-335 nm at 10 nm/s with an excitation wavelength of 295 nm and slits corresponding to a spectral width of 2 nm.

Circular Dichroism (CD). CD analysis was carried out at 0.57 mg protein/mL in a 0.1 mm pathlength quartz cuvette (Starna, Hainault, UK) with a Jasco J-815 spectrometer (Jasco, Easton, MD, USA) at 20 ºC. Spectra from 260-190 nm were recorded at 50 nm/min in 1 nm steps with a band width setting of 1 nm. Spectra were smoothed using the binomial function included in the Jasco spectra analysis software package (version 1.54.03). As reported previously[7], high concentrations of urea give noisy CD data in the 190-215 nm range. This region was omitted from spectra of urea-containing samples in the current study.

The α-helical content of DgkA was calculated using the relationship: $\alpha\text{-helix}\ (\%) = ([\theta]_{222} - 3{,}000) \times 100/(-36{,}000 - 3{,}000)$ where $[\theta]_{222}$ corresponds to Mean residue ellipticity at 222 nm[8].

Rounds of reconstitution. The solubility of DgkA* in acidic urea was only 1 mg/mL. After reconstitution under standard conditions, the protein concentration in the cubic phase would only reach 0.4 mg/mL, 12 - 15 times below the concentration used typically for crystallization (4.8 mg/mL in monoolein; 6 mg/mL in 7.8 MAG)[9]. To incrementally raise protein concentration in the mesophase a method referred to as ‘rounds of reconstitution’ was implemented, as outlined below (**Fig. S2**)).

Sixteen microliters of DgkA* at 1 mg/mL in acidic urea was homogenized with 24 μL of monoolein (**Step 1, Fig. S2**) by using a coupled syringe mixer consisting of two 0.1 mL Hamilton syringes (Syringes A and B) and a narrow-bore coupler[10]. After mixing, the freshly

formed protein-laden mesophase was transferred to Syringe A. Syringe B was replaced with a 0.5 mL Hamilton syringe (Syringe C) containing 0.45 mL **Refolding Buffer** (1 mM TCEP, 0.1 M NaCl, 0.1 M HEPES pH 7.5) and the mesophase in Syringe B was transferred to Syringe C. Empty Syringe A was replaced with an empty 0.5 mL syringe (Syringe D) and the contents of the coupled syringe device were mixed at RT to dilute out the denaturant and to reconstitute and refold the protein (**Step 2**). After 30 min of mixing, the contents were transferred to Syringe D and the coupled syringe was left to sit at RT for 10 min in a vertical position with Syringe D on top. The mesophase rises in the barrel of Syringe D to the Teflon tip of the plunger where it naturally sticks together. As a result, the lower, phase-separated buffer fraction could be transferred from Syringe D into Syringe C with very little loss of mesophase. Syringe C was detached and excess buffer discarded (**Step 3**). The process of removing excess buffer was repeated until the volume of mesophase with residual buffer was less than 0.1 mL. At this point, the contents were transferred to coupled 0.1 mL syringes (Syringes E, F) to further reduce excess buffer. The volume recorded at this final stage was ~38 μL with a calculated protein concentration in the mesophase of 0.4 mg/mL. The entire contents were transferred to Syringe F in preparation for rounds of reconstitution.

Rounds of reconstitution began by mixing 50-60 μL of DgkA at 1 mg/mL in acidic urea with the ~38 μL of protein-laden mesophase obtained from the initial reconstitution step above (**Step 4**). This volume ratio ensures that the urea concentration does not drop below 6.5 M at which point the protein may come out of solution. For this purpose, the protein solution and mesophase were contained in coupled 0.1 mL syringes (Syringes F, G). Mixing was carried out for 30 min after which the contents were divided equally between the two syringes. The contents of each were washed three times with Refolding Buffer, as outlined above, and finally combined in one syringe (**Steps 5 and 6**). Rounds of reconstitution (**Steps 4 – 6**) were repeated until a total of 200-320 μL (0.20 - 0.32 mg) of DgkA in acidic urea solution had been used. It is estimated that 1.5-2 μL of mesophase was lost at each round. The protein-laden mesophase prepared in this way contained a small volume of excess buffer and was cloudy (**Step 7**). To absorb excess buffer and to convert the system entirely to the optically clear mesophase ready for crystallization and functional assays, 2-3 mg of monoolein was mixed with the sample (**Step 8**).

After 3-5 rounds of reconstitution, ~32 μL of mesophase with an estimated DgkA concentration of 6-9 mg/mL was recovered. It was washed three times with 0.45 mL **Refolding Buffer** and then three times with **Washing Buffer** (1 mM TCEP, 0.1 M NaCl, 10 mM Tris pH 7.8) to remove residual formic acid and urea and to switch from HEPES to Tris-HCl buffer in preparation for enzyme assays and for crystallization trials.

The rounds of reconstitution method was implement also with 7.8 MAG in which case equal volumes of lipid and protein solution were used to form the mesophase initially, following an established protocol[9]. The entire process results in ~28 and ~22 μL of protein-laden mesophase for monoolein and 7.8 MAG, respectively.

***In meso* crystallization.** All crystallization trials were carried out at 4 °C with an *in meso* robot[11] using protein-laden mesophase obtained following the rounds of reconstitution protocol. WT DgkA in monoolein was crystallized using a precipitant containing 0.1 M NaCl, 0.1 M $LiNO_3$, 7-10 %(v/v) MPD, 0.1 M $Na_3C_6H_5O_7$ (sodium citrate) pH 5.6. The precipitant used with 7.8 MAG included 0.1 M NaCl, 0.1 M $LiNO_3$, 3-6 %(v/v) MPD, 60 mM $Mg(CH_3COO)_2$ (magnesium acetate), 50 mM $Na_3C_6H_5O_7$ pH 5.6. The themostable DgkA mutant was crystallized in 7.8 MAG with 0.1 M NaCl, 3-6 % (v/v) MPD, 60 mM $Mg(CH_3COO)_2$, 50 mM $Na_3C_6H_5O_7$. Crystals were harvested and snap-cooled as described[12].

X-ray diffraction. Diffraction data were collected on GM/CA CAT beamline 23ID-B, the Advanced Photon Source (APS), beamline I24, the Diamond Light Source (DLS), and PX II at the Swiss Light Source (SLS). At the APS, data were collected with a 1° oscillation and a 1 s exposure per image, a collimated beam size of 10 × 10 μm^2 and a sample-to-detector distance of 350-500 mm, with a MAR 300 CCD detector using 1.033 Å wavelength X-rays. At the DLS, data were collected with a 0.2 ° oscillation and a 0.2 s exposure per image, a micro-focus beam size of 10 × 10 μm^2 and a sample-to-detector distance of 400-650 mm, with a Pilatus 6M detector using 0.978 Å wavelength X-rays. At the SLS, data were collected with a 0.1 ° oscillation and 0.1 s exposure per image, a collimated beam size of 10 or 30 × 15 μm^2 and a sample-to-detector distance of 300-490 mm, with a Pilatus 6M detector using 1.033 Å wavelength X-rays. Diffraction images, recorded with a 10-fold attenuated beam, were used to locate crystals in the mesophase and to center on highly ordered regions of the crystal. Complete data sets for wild-type and thermostable DgkA* were collected with single crystals grown in the 7.8 MAG mesophase. Data was reduced with xia2[13] using XDS[14], XSCALE and SCALA[15].

Structure solution and refinement. Initial phases for thermostable and wild-type DgkA* were obtained by molecular replacement using Phaser[16] with the protein component of a published structures (PDB 3ZE3 and 3ZE4, respectively)[2] as the search model. In subsequent cycles of iterative model building and refinement, the program Coot[17] was used for model building; the program Phenix[18] was used for refinement. For the low resolution wild-type DgkA* structure at 3.8 Å, non-crystallographic symmetry restraints were applied to torsion angles during refinement. Structures were visualised with Pymol[19].

Table S1| Data collection and refinement statistics for crystals of refolded DgkA.

**Data collection**	Thermostable DgkA (PDB entry 4BRB)	Wild-type DgkA (PDB entry 4UP6)
Space group	$P2_12_12_1$	$P3_121$
Cell dimensions		
a, b, c (Å)	75.50, 91.57, 143.57	75.39, 75.39, 197.20
α, β, γ (°)	90, 90, 90	90, 90, 120
Wavelength (Å)	1.03319	1.03320
Beamline	PX II (X10SA), SLS	ID-23-B, APS
Number of Crystals	1	1
Resolution (Å)	71.79-2.55 (2.62-2.55)*	54.44-3.80 (3.90-3.80)*
R_{merge}	0.120 (1.001)*	0.135 (1.130)*
R_{pim}	0.056 (0.463)*	0.053 (0.423)*
$I / \sigma I$	7.9 (2.2)*	9.2 (1.7)*
Completeness (%)	98.3 (97.9)*	95.5 (97.8)*
Redundancy	5.5 (5.1)*	6.4 (6.5)*
Wilson B-factor (Å^2)	55.99	136.72
**Refinement**		
Resolution (Å)	45.23-2.55	46.32 - 3.80
Number of reflections	32,531	6,490
R_{work} / R_{free}	0.2173 / 0.2614	0.3245 / 0.3715
No. atoms	4,762	2,472
Protein	4,503	2,472
Ligand / ion	238	0
Water	21	0
Number of chains	6	3
Number of residues	593	323
Chains (residues present)	A (6-120), B (23-121), C (29-121), D (14-121), E (35-120), F (29-120)	A (7-120), B (4-120), C (28-119)
B-factors (Å^2)	66.43	145.86
Protein	65.43	145.86
Ligand / ion	85.9	n/a
Water	60.31	n/a
R.m.s deviations		
Bond lengths (Å)	0.01	0.004
Bond angles (°)	1.06	0.838
Ramachandran plot		
Favoured region	98.45	98.11
Allowed region	1.55	1.89
Outlier region	0.0	0.0
MolProbity clash score	9.74	14.81

*Highest resolution shell is shown in parenthesis.

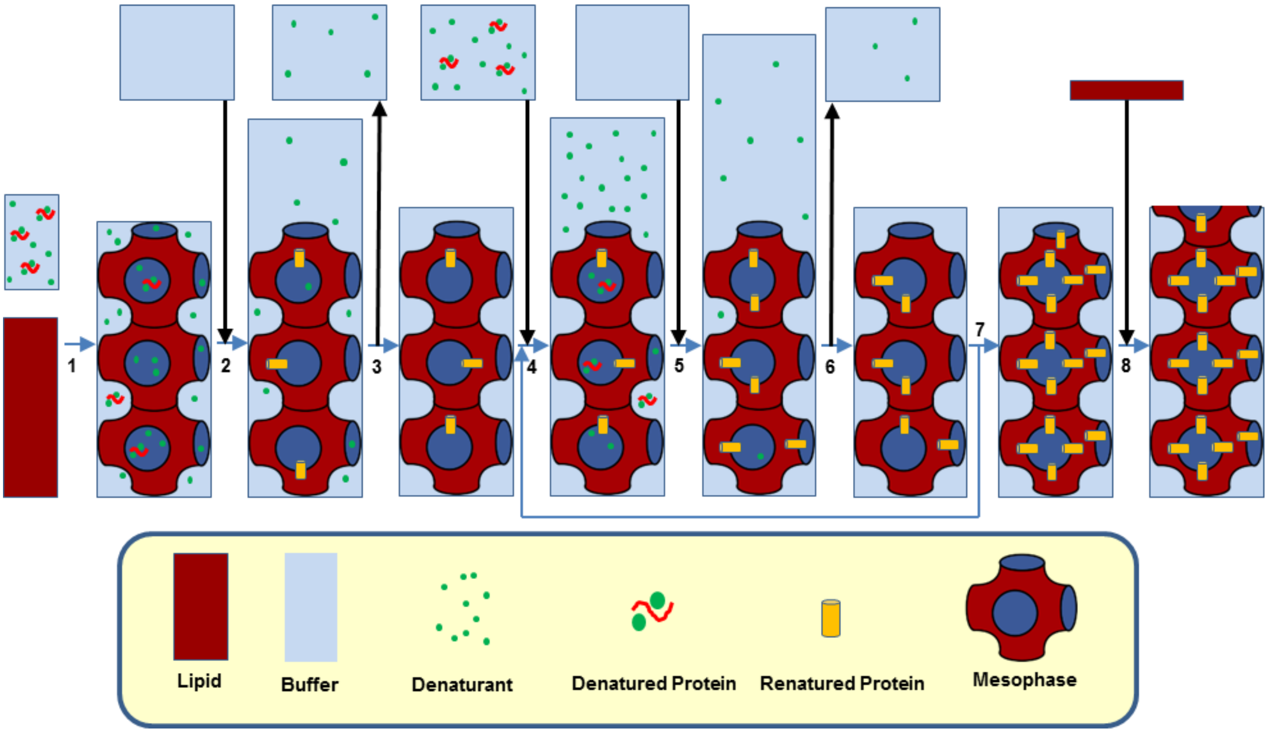

Fig. S1. Membrane protein renaturation in the lipid cubic phase and how protein concentration in the mesophase can be increased by sequential rounds of reconstitution. **Step 1:** Monoacylglycerol lipid and solubilized membrane protein in denaturant solution are mixed, spontaneously forming the bicontinuous cubic mesophase. **Step 2:** Denaturant-free buffer is added which enables denaturant to flood out of the nanoporous mesophase and the protein to reconstitute and renature in the bilayer of the mesophase. **Step 3:** Most of the excess denaturant-containing buffer, that naturally separates from the mesophase, is removed. When enough protein has been reconstituted into the mesophase for end use, the process moves to **Step 8** where excess buffer is absorbedd by mixing in a small amount of lipid to produce homogenous, optically clear cubic phase. **Step 4:** When insufficient protein has been incorporated into the mesophase, rounds of reconstitution begins by mixing in fresh denaturant solution that contains solubilized membrane protein. The protein preferentially associates with the mesophase. **Step 5:** Denaturant-free buffer is added to dilute out the denaturant thereby facilitating reconstitution and renaturation of solubilized membrane protein. **Step 6:** Most of the excess denaturant-containing buffer that naturally separates from the mesophase is removed leaving behind mesophase enriched in membrane protein. **Step 7:** Steps 4, 5 and 6 are repeated sequentially until sufficient protein has been reconstituted and renatured in the mesophase. **Step 8:** Excess buffer is absorbedd by mixing in a small amount of lipid to produce homogenous, optically clear protein-laden cubic phase suitable for end use.

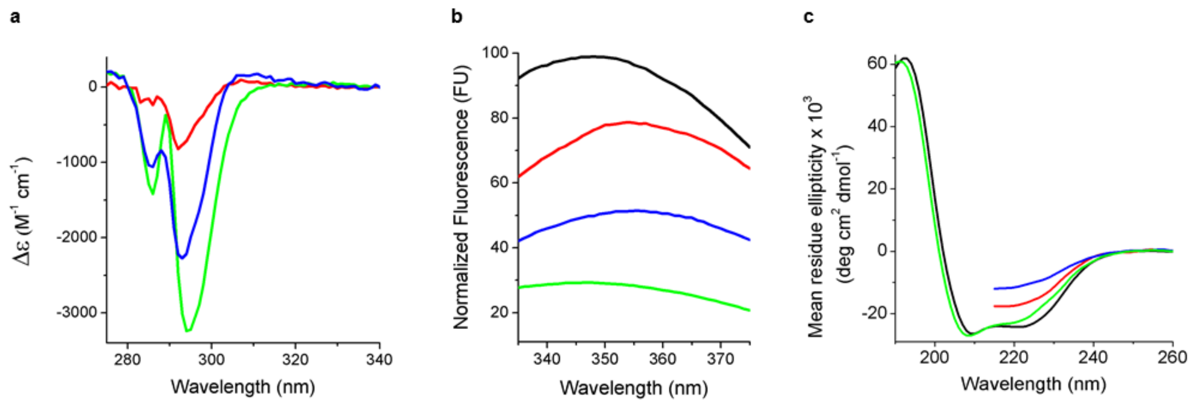

Fig. S2. Spectroscopic evidence for denatured wild-type DgkA. **a**, UV difference spectra. The reference spectrum was recorded with untreated WT DgkA in detergent micelles. Sample spectra were recorded with detergent-free WT DgkA* in SDS (green), in acidic urea (blue), and DgkA in acidic urea containing detergent (red) and subtracted from the reference spectrum. The minimum at 294 nm is characteristic of denatured DgkA[6]. **b**, Fluorescence emission of untreated WT DgkA in detergent micelles (black) and in acidic urea containing detergent (red), and of detergent-free WT DgkA* in SDS (green) and in acidic urea (blue). A red shift, coupled with a reduction in fluorescence yield, is indicative of tryptophan exposure to a polar environment and to denaturation. **c**, Circular dichroism spectra of untreated WT DgkA in detergent micelles (black) and in acidic urea containing detergent (red), and of detergent-free WT DgkA* in SDS (green) and in acidic urea (blue). In urea, data are only shown to 215 nm due to strong absorbance at lower wavelengths. A strong negative ellipticity at 222 nm correlates with α-helical secondary structure and the folded state. All data shown were collected with WT DgkA. The corresponding data for thermostable DgkA are shown in **Fig. 2**. Both constructs behaved similarly.

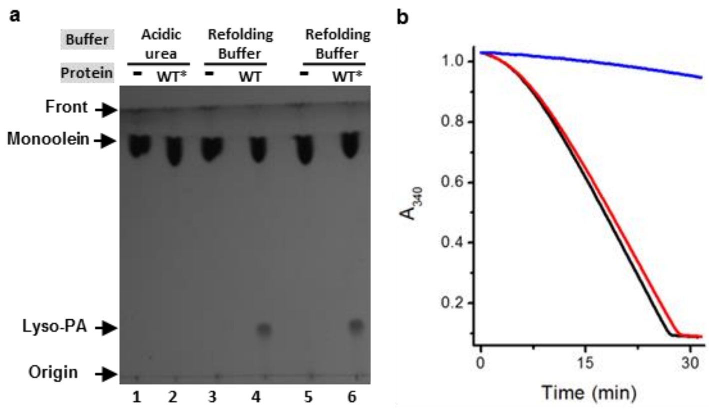

Fig. S3. Wild-type DgkA renatured in the cubic phase is fully functional. a, Thin layer chromatographic analysis shows that equal amounts of product, lysophosphatidic acid (lyso-PA), were formed after 1 hour of reaction catalyzed by untreated WT DgkA (lanes 3, 4) and by detergent-free WT DgkA* (lanes 5, 6) renatured from acidic urea with denaturant-free refolding buffer. No reaction is seen when acidic urea remained in the mesophase (lanes 1, 2). **b**, Kinase progress curves for untreated WT DgkA (black) and detergent-freeWT DgkA renatured from acidic urea (red). A protein-free control is shown (blue).

References

1. Li, D. & Caffrey, M. Lipid cubic phase as a membrane mimetic for integral membrane protein enzymes. *Proc. Natl. Acad. Sci. U. S. A.* **108**, 8639 (2011).
2. Li, D. *et al.* Crystal structure of the integral membrane diacylglycerol kinase. *Nature* **497**, 521 (2013).
3. Urbani, A. & Warne, T. A colorimetric determination for glycosidic and bile salt-based detergents: applications in membrane protein research. *Anal. Biochem.* **336**, 117 (2005).
4. Rouser, G., Fkeischer, S. & Yamamoto, A. Two dimensional then layer chromatographic separation of polar lipids and determination of phospholipids by phosphorus analysis of spots. *Lipids* **5**, 494 (1970).
5. Badola, P. & Sanders, C. R. Escherichia coli diacylglycerol kinase is an evolutionarily optimized membrane enzyme and catalyzes direct phosphoryl transfer. *J. Biol. Chem.* **272**, 24176 (1997).
6. Lau, F. W. & Bowie, J. U. A method for assessing the stability of a membrane protein. *Biochemistry* **36**, 5884 (1997).
7. Dutta, A., Tirupula, K. C., Alexiev, U. & Klein-Seetharaman, J. Characterization of membrane protein non-native states. 1. Extent of unfolding and aggregation of rhodopsin in the presence of chemical denaturants. *Biochemistry* **49**, 6317 (2010).
8. Barrow, C. J., Yasuda, A., Kenny, P. T. & Zagorski, M. G. Solution conformations and aggregational properties of synthetic amyloid beta-peptides of Alzheimer's disease. Analysis of circular dichroism spectra. *J. Mol. Biol.* **225**, 1075 (1992).
9. Li, D., Shah, S. T. & Caffrey, M. Host Lipid and Temperature as Important Screening Variables for Crystallizing Integral Membrane Proteins in Lipidic Mesophases. Trials with Diacylglycerol Kinase. *Cryst. Growth Des.* **13**, 2846 (2013).
10. Cheng, A., Hummel, B., Qiu, H. & Caffrey, M. A simple mechanical mixer for small viscous lipid-containing samples. *Chem. Phys. Lipids* **95**, 11 (1998).
11. Li, D., Boland, C., Walsh, K. & Caffrey, M. Use of a robot for high-throughput crystallization of membrane proteins in lipidic mesophases. *J. Vis. Exp.* **67**, e4000 (2012).
12. Li, D., Boland, C., Aragao, D., Walsh, K. & Caffrey, M. Harvesting and cryo-cooling crystals of membrane proteins grown in lipidic mesophases for structure determination by macromolecular crystallography. *J. Vis. Exp.* **67**, e4001 (2012).
13. Winter, G., Lobley, C. M. & Prince, S. M. Decision making in xia2. *Acta Crystallogr. D* **69**, 1260 (2013).
14. Kabsch, W. Xds. *Acta Crystallogr. D* **66**, 125 (2010).
15. Evans, P. Scaling and assessment of data quality. *Acta Crystallogr. D* **62**, 72 (2006).
16. McCoy, A. J. *et al.* Phaser crystallographic software. *J. Appl. Crystallogr.* **40**, 658 (2007).
17. Emsley, P. & Cowtan, K. Coot: model-building tools for molecular graphics. *Acta Crystallogr. D* **60**, 2126 (2004).
18. Adams, P. D. *et al.* PHENIX: a comprehensive Python-based system for macromolecular structure solution. *Acta Crystallogr. D* **66**, 213 (2010).
19. PyMOL. The PyMOL Molecular Graphics System, Version 1.2r2 Schrödinger, LLC.
